# Draft Genome Sequences of Avian Chlamydia abortus Genotype G2 Strain 15-49d3, Isolated from Mallard, and Genotype 1V Strain 15-58d44, Isolated from Magpie in Poland

**DOI:** 10.1128/MRA.01203-20

**Published:** 2021-04-08

**Authors:** Kinga Zaręba-Marchewka, Monika Szymańska-Czerwińska, Krzysztof Niemczuk

**Affiliations:** a Department of Cattle and Sheep Diseases, National Veterinary Research Institute, Puławy, Poland; b Laboratory of Serological Diagnosis, National Veterinary Research Institute, Puławy, Poland; University of Maryland School of Medicine

## Abstract

Here, we report the draft genome sequences of avian Chlamydia abortus genotype G2 strain 15-49d3, isolated from mallard, and genotype 1V strain 15-58d44, isolated from magpie in Poland. The total genome assembly lengths are 1,140,139 bp and 1,158,207 bp, respectively.

## ANNOUNCEMENT

Chlamydiae are Gram-negative bacteria distributed worldwide in humans, livestock, companion animals, and free-living animals (1–3). The genus *Chlamydia* includes 14 characterized species (4–7) and 4 candidate species (8–11). C. abortus is a cause of abortion and fetal loss in ruminants and other mammals, while C. psittaci is an etiological agent of avian chlamydiosis in birds (12, 13). Both of them are known to be associated with zoonotic potential, thus posing a threat to human health (14, 15). Recently, the occurrence of *C. abortus* was noted in nonmammalian hosts (16, 17). Novel strains originating from wild birds were provisionally named avian *C. abortus* (17). Considering the worldwide distribution of these strains in diverse bird families, avian *C. abortus* strains should be considered epidemiologically relevant (17–20).

Here, we present the draft genome sequences of avian Chlamydia abortus genotype G2 strain 15-49d3, isolated from mallard, and genotype 1V strain 15-58d44, isolated from magpie. Molecular analysis based on multilocus sequence typing (MLST) performed in our previous study clearly classified these isolates within the *C*. *abortus* species (17).

Avian *C. abortus* strains representing genotypes G2 and 1V were isolated from cloacal swab samples originating from mallard and magpie on Buffalo green monkey (BGM) cell culture with UltraMDCK serum-free medium (Lonza Cologne, Germany). The medium was renewed after 18 h. Cultures were propagated in T25 flasks and incubated at 37°C with 5% CO_2_ in a fully humidified cabinet for 72 h (17). After three passages, cell culture was used for DNA extraction using a QIAamp DNA minikit (Qiagen, Germany) following the manufacturer’s instructions. Host DNA was removed using a NEBNext microbiome DNA enrichment kit (New England Biolabs, USA). Genomic libraries were constructed using a Nextera XT DNA library preparation kit and a Nextera XT index kit (Illumina, USA). Sequencing was performed on a MiSeq sequencer (Illumina) with the 2 × 300-bp paired-end protocol. The raw read quality was evaluated based on FastQC 0.1.18 (21), followed by adapter and quality trimming using Trimmomatic 0.36 (22). Nonchlamydial reads originating from host DNA were identified through mapping against the African green monkey genome using the Burrows-Wheeler Aligner MEM algorithm (BWA-MEM) 0.7.15 (23) and discarded. The total numbers of filtered reads of strains 15-49d3 and 15-58d44 were 172,095 (43,895,761 bp) and 619,570 (148,956,163 bp), respectively. Trimmed and filtered reads were assembled using SPAdes 3.11.1 (24). The coverage cutoff was set at 10× and 500-bp length, which resulted in 2 contigs (15-49d3) and 5 contigs (15-58d44). The lengths of the total genome assemblies of strains 15-49d3 and 15-58d44 were 1,140,139 bp and 1,158,207 bp, respectively. The *N*_50_ value for strain 15-58d44 was 776,426 bp. The draft genome sequences were annotated using the Rapid Annotations using Subsystems Technology (RAST) pipeline (25). Detailed properties of the genomes are presented in Table 1. Default parameters were used for all software unless otherwise specified.

**TABLE 1:** Genome properties of avian Chlamydia abortus genotype G2 strain 15-49d3 and genotype 1V strain 15-58d44

Feature^*a*^	Value	% of total	Accession no.
15-49d3
Chromosome
No. of contigs	1
Length (bp)	1,132,456
Scaffold 1 (bp)	1,132,456	LS450956
Avg read coverage (×)	∼37.5
DNA coding (bp)	1,007,970	89.01
DNA G+C content (%)	39.6
No. of genes
Total	1,089	100
Protein coding genes	1,048	96.24
RNA genes	41	3.76
Pseudogenes
Genes with functional prediction	750	68.87
Genes assigned to COGs	497	45.64
Genes with Pfam domains	753	69.15
Genes with signal peptides	52	4.78
Genes with transmembrane helices	234	21.49
Plasmid		
No. of contigs	1
Length (bp)	7,683
Scaffold 2 (bp)	7,683	LS450957
Avg read coverage (×)	∼102.5
DNA G+C content (%)	32.6
No. of CDS	8
15-58d44
Chromosome
No. of contigs	4
Total length (bp)	1,150,527
Scaffold 1 (bp)	776,426	CAJHTZ010000001
Scaffold 2 (bp)	372,709	CAJHTZ010000002
Scaffold 4 (bp)	870	CAJHTZ010000004
Scaffold 5 (bp)	522	CAJHTZ010000005
Avg read coverage (×)	∼76.38
DNA coding (bp)	1,028,907	89.43
DNA G+C content (%)	39.9
No. of genes
Total genes	1,080	100
Protein coding genes	1,039	96.20
RNA genes	41	3.80
Pseudogenes
Genes with functional prediction	710	65.74
Genes assigned to COGs	500	46.30
Genes with Pfam domains	764	70.74
Genes with signal peptides	57	5.28
Genes with transmembrane helices	257	23.80
Plasmid		
No. of contigs	1
Length (bp)	7,680
Scaffold 3 (bp)	7,680	CAJHTZ010000003
Avg read coverage (×)	∼304.5
DNA G+C content (%)	33.0
No. of CDS	8

a CDS, coding DNA sequences; COG, clusters of orthologous groups.

### Data availability.

The genome sequences of strain 15-49d3 have been deposited at ENA/GenBank/DDBJ under the accession numbers LS450956 and LS450957, and the raw reads under number ERS2484025. The genome sequences of strain 15-58d44 have been deposited at ENA/GenBank/DDBJ under the accession numbers CAJHTZ010000001, CAJHTZ010000002, CAJHTZ010000003, CAJHTZ010000004, and CAJHTZ010000005, and the raw reads have been deposited under SRA number ERS2484027. The BioProject accession number is PRJEB26715.
